# *Arabidopsis* DEFECTIVE KERNEL1 regulates cell wall composition and axial growth in the inflorescence stem

**DOI:** 10.1002/pld3.27

**Published:** 2017-12-06

**Authors:** Dhika Amanda, Monika S. Doblin, Colleen P. MacMillan, Roberta Galletti, John F. Golz, Antony Bacic, Gwyneth C. Ingram, Kim L. Johnson

**Affiliations:** ^1^ Max Planck Institute for Plant Breeding Research Köln Germany; ^2^ ARC Centre of Excellence in Plant Cell Walls School of BioSciences The University of Melbourne Parkville VIC Australia; ^3^ CSIRO Agriculture and Food Canberra ACT Australia; ^4^ Laboratoire Reproduction et Développement des Plantes Université de Lyon CNRS INRA UCB Lyon 1 Lyon France; ^5^ School of BioSciences The University of Melbourne Parkville VIC Australia

**Keywords:** biomechanics, CALPAIN, cell wall, DEK1, stem

## Abstract

Axial growth in plant stems requires a fine balance between elongation and stem mechanical reinforcement to ensure mechanical stability. Strength is provided by the plant cell wall, the deposition of which must be coordinated with cell expansion and elongation to ensure that integrity is maintained during growth. Coordination of these processes is critical and yet poorly understood. The plant‐specific calpain, DEFECTIVE KERNEL1 (DEK1), plays a key role in growth coordination in leaves, yet its role in regulating stem growth has not been addressed. Using plants overexpressing the active CALPAIN domain of DEK1 (*CALPAIN OE)* and a *DEK1* knockdown line (*amiRNA‐DEK1*), we undertook morphological, biochemical, biophysical, and microscopic analyses of mature inflorescence stems. We identify a novel role for DEK1 in the maintenance of cell wall integrity and coordination of growth during inflorescence stem development. *CALPAIN OE* plants are significantly reduced in stature and have short, thickened stems, while *amiRNA‐DEK1* lines have weakened stems that are unable to stand upright. Microscopic analyses of the stems identify changes in cell size, shape and number, and differences in both primary and secondary cell wall thickness and composition. Taken together, our results suggest that DEK1 influences primary wall growth by indirectly regulating cellulose and pectin deposition. In addition, we observe changes in secondary cell walls that may compensate for altered primary cell wall composition. We propose that DEK1 activity is required for the coordination of stem strengthening with elongation during axial growth.

## 1 INTRODUCTION

A single, highly conserved *DEFECTIVE KERNEL1* (*DEK1*) gene is found in all land plants (Ahn, Kim, Lim, Kim, & Pai, 2004; Margis & Margis‐Pinheiro, 2003; Ono & Sorimachi, 2012; Wang et al., 2003). DEK1 has a predicted structure that includes 21–24 transmembrane domains at the N‐terminus, a juxtamembrane domain, and a cytoplasmic CALPAIN‐like domain at the C‐terminus (Becraft, Li, Dey, & Asuncion‐Crabb, 2002; Johnson, Faulkner, Jeffree, & Ingram, 2008; Liang et al., 2013; Wang et al., 2003). The mechanism of DEK1 activation is likely to be similar to that of the animal cytoplasmic CALPAINs, which undergo calcium‐dependent autolytic cleavage of the N‐terminus, leading to enzyme activation and initiation of downstream signaling events (García Díaz, Gauthier, & Davies, 2006). This is supported by experiments showing the CALPAIN domain alone, from either *Arabidopsis*,* Physcomitrella* or maize DEK1, can complement the *Arabidopsis dek1* mutant (Johnson et al., 2008; Liang et al., 2013; Perroud et al., 2014). These experiments suggest the catalytic CALPAIN domain of DEK1 is functionally conserved in land plants from mosses to angiosperms and likely arose early in land plant evolution (Liang et al., 2013).

The role of DEK1 in plants has been best characterized in *Arabidopsis* (Galletti et al., 2015; Johnson, Degnan, Ross Walker, & Ingram, 2005; Johnson et al., 2008; Roeder, Cunha, Ohno, & Meyerowitz, 2012). DEK1 is crucial for early embryo development as *dek1* loss‐of‐function mutants are embryo‐lethal (Johnson et al., 2005; Lid et al., 2005). Only the use of an artificial microRNA‐mediated approach to reduce *DEK1* expression levels and the isolation of the weak *dek1‐4* allele have enabled investigation of its function postembryonically. These studies suggest that an important role for DEK1 is in the specification and maintenance of the epidermis (Ahn et al., 2004; Galletti et al., 2015; Lid et al., 2002; Roeder et al., 2012). Changes in epidermal cell size and shape are observed in *dek1‐4* plants, including a near absence of giant cells in sepals (Roeder et al., 2012) and the production of less complex and more homogeneously sized pavement cells in cotyledons (Galletti et al., 2015). Reduced lobing in cotyledon pavement cells in *p35S:amiDEK1* lines and decreased expression of several epidermis‐specific transcription factors suggest that DEK1 specifically promotes the differentiation and maintenance of epidermal identity (Galletti et al., 2015).

Interestingly, the expression of *DEK1* is not restricted to the epidermal layer and is detected in all cell types throughout development (Johnson et al., 2005; Liang, Brown, Fletcher, & Opsahl‐Sorteberg, 2015; Lid et al., 2005). Although the epidermis appears to be most sensitive to changes in DEK1 levels, phenotypes in underlying cell layers have been observed (Ahn et al., 2004; Johnson et al., 2008). Silencing of the *Nicotiana benthamiana DEK1* gene through virus‐induced gene silencing (VIGS) resulted in changes to mesophyll cell shape and increased numbers of cells in stems, leading to the suggestion that DEK1 plays a role in regulating the balance between cell division and cell expansion (Ahn et al., 2004). Phenotypes in plants overexpressing the CALPAIN domain of DEK1 (*CALPAIN OE*) in *Arabidopsis* also support a role in the regulation of cell division and expansion, as leaves show excess growth in all cell layers (Johnson et al., 2008). The epidermal layer is thought to regulate organ growth non‐cell‐autonomously by sending signals to underlying layers (Ingram & Waites, 2006; Savaldi‐Goldstein & Chory, 2008; Takada & Iida, 2014). Growth coordination is crucial both to the generation of flat, blade‐like organs such as leaves, and radial upright organs such as the stem (Maeda et al., 2014; Nath, Crawford, Carpenter, & Coen, 2003; Palatnik et al., 2003). However, although DEK1 has been proposed to play a role in coordinating growth within and between cell layers (Becraft et al., 2002; Johnson et al., 2008), studies of DEK1 function have largely been confined to leaf‐like organs with very little known about its role in tissues that provide mechanical support, such as those present in the stem.

Plants support themselves during aerial growth due to the mechanical strength provided by the plant cell wall. Plant cell walls can be divided into primary and secondary walls, with primary walls being thin and flexible, features that enable growth of cells while maintaining considerable tensile strength (Bacic, Harris, & Stone, 1988). These characteristics are critical for the effective harnessing of cell turgor pressure, which is the main factor responsible both for driving cellular growth and for supporting the upright stance of young plant tissues (Cosgrove & Jarvis, 2012; Schopfer, 2006). Secondary walls are thickened structures, often containing lignin, and are produced by specific cell types (Keegstra, 2010; Kumar, Campbell, & Turner, 2016). These walls not only act to support the plants own weight, by providing resistance to compression and bending, but also resist external mechanical stresses and hydraulic pressure gradients arising from movement of water through the vasculature (Cosgrove, 1986; Cosgrove & Jarvis, 2012). In tissues destined to produce secondary walls, including xylem and interfascicular fibers, deposition of cell wall components must be developmentally regulated to ensure that cell expansion and shoot growth are coordinated (Albersheim, Darvill, Roberts, Sederoff, & Staehelin, 2011; Raven, Evert, & Eichhorn, 2005). A relationship between secondary cell wall deposition and stem growth must exist and likely involves mechanical sensing feedback mechanisms that ensure tissue integrity is maintained.

Components of primary cell walls, such as cellulose, xyloglucans, heteroxylans, and more recently pectic polysaccharides, have also been shown to provide mechanical support to developing stems (Hongo, Sato, Yokoyama, & Nishitani, 2012; Zhu et al., 2015). Identification of *irregular xylem* (*irx*) mutants, which have decreased stem stiffness due to reduced levels of cellulose, suggests that this polymer plays an important role in determining stem properties. Cellulose microfibrils are embedded and cross‐linked in a matrix phase composed of noncellulosic polysaccharides (often referred to as hemicelluloses) and pectins. Recent NMR studies show that pectins, rather than xyloglucans, make the majority of contacts with cellulose and might serve as mechanical tethers between cellulose microfibrils to form a strong and extensible network (Wang, Zabotina, & Hong, 2012). A “biomechanical hotspots” wall model integrates these data and suggests cellulose–pectin interactions are prevalent and xyloglucan makes contact with cellulose at limited sites that are important for cell wall integrity (Cosgrove, 2014). This view is supported by studies showing that pectin influences the biomechanical properties of both primary and secondary walls (Goulao, Vieira‐Silva, & Jackson, 2011; Hongo et al., 2012; Mellerowicz & Gorshkova, 2011; Siedlecka et al., 2008).

In this study, we investigate the role of DEK1 in inflorescence stem development. We show that CALPAIN OE lines develop a short and thickened stem compared to wt plants, whereas lines with reduced *DEK1* activity have a mechanically weakened stem resulting in a prostrate stem phenotype. We establish a role for DEK1 in regulating cell wall pathways leading to changes in cell wall composition and organization, cell size and shape, composition of secondary walls, and ultimately stem growth.

## 2 MATERIALS AND METHODS

### 2.1 Plant material

Wild type (wt; *Arabidopsis thaliana* Columbia‐0 ecotype), plants constitutively overexpressing *CALPAIN* (*CALPAIN OE*) (Johnson et al., 2008), and plants constitutively expressing an artificial microRNA targeting the *DEK1* transcript (*amiRNA‐DEK1*) and the *dek1‐4* allele in Columbia‐0 (Galletti et al., 2015; Roeder et al., 2012) were grown under short‐day conditions with 8‐hr light/16‐hr dark cycle at 21°C for 12 weeks.

### 2.2 Phenotypic and biomechanical characterization of stems

The height of the main stem was measured in 4‐month‐old plants at growth stage 6.50–6.90, when the stem inflorescence growth reaches >80% of its final height (Boyes et al., 2001). Measurement of stem diameter and Maule staining (Sibout et al., 2005) were performed on hand sections of fresh tissue at the stem base and 3 cm above the stem base. Cross sections were imaged on a Leica M205A dissecting microscope (Leica Microsystems, Germany).

Tensile and three‐point flexural tests were performed using a 4500 series Instron universal testing machine (series IX automated materials testing system, http://www.instron.co.uk) with nine biological replicates as outlined in MacMillan, Mansfield, Stachurski, Evans, and Southerton (2010). Biomechanical tests include cross‐sectional area as an input during each test. The cross‐sectional area is an average of two perpendicular measurements of the stem at the point where the tests occur for compression.

### 2.3 Cryo‐Scanning Electron Microscope (Cryo‐SEM)

A 1.5‐mm piece of freshly cut stem material was cryo‐preserved and its wax crystallization pattern viewed on a Quanta E SEM (FEI, USA).

### 2.4 Fixation and embedding of tissue for immunolocalization

The fixation protocol for *Arabidopsis* tissue was adapted from Wilson and Bacic (2012). Tissue (0.5 cm) at the base of the inflorescence stem was fixed in 2.5% (v/v) glutaraldehyde in potassium phosphate buffer (0.025 M PBS, pH 7) and then embedded in 100% LR white resin. Thin sections (90 nm and 250 nm) were obtained with a Leica Ultracut R microtome (Leica Microsystems, Germany) and placed on formvar‐coated 100‐mesh gold grids (Proscitech, Australia) for TEM or glass microscope slides for fluorescence immunolocalization experiments.

### 2.5 Antibodies

Antibodies used to recognize the following cell wall epitopes: homogalacturonan (HG) (JIM5 and JIM7; Knox, Linstead, King, Cooper, & Roberts, 1990), type I galactan (LM5; Jones, Seymour, & Knox, 1997), arabinan (LM6; Willats, Marcus, & Knox, 1998), xyloglucan (LM15; Marcus et al., 2008), and cellulose‐directed CBM (CBM3a; Blake et al., 2006) were obtained from PlantProbes (UK). For fluorescence microscopy, either fluorescein isothiocyanate (FITC)‐conjugated anti‐rat IgG (Sigma‐Aldrich; #F1763) or Alexa Fluor 488 goat anti‐mouse IgG (H+L) (Life Technology; #A11001) was used as the secondary antibody. For TEM studies, 18‐nm colloidal gold conjugated to either goat anti‐rat or anti‐mouse IgG (both Jackson ImmunoResearch) was used.

Specificity of pectin labeling was assessed by pre‐adsorbing JIM5 with pectin DE 30% substrate (1 mg/ml; CP Kelco Aps) and JIM7 with pectin DE 60% substrate (1 mg/ml; Herbstreith & Fox KG #01401094) overnight.

### 2.6 Demasking of cell wall epitopes

The method for epitope demasking was modified from Wilson et al. (2015). Samples were pretreated with 2 M urea in PBS for 30 mins, followed by five washes in PBS. Each section was pretreated with xyloglucanase (1 U/ml; Megazyme #EC 3.2.1.151) in PBS containing 2 M urea and 0.1% Tween‐20 for 4.5 hr at room temperature to remove xyloglucan from cell walls (Xue, Bosch, & Knox, 2013). After washing thoroughly six times with 2 M urea with 0.1% Tween‐20 in PBS, and three times with PBS, immunolabeling was carried out following the protocol from Coimbra, Almeida, Junqueira, Costa, and Pereira (2007) for immunofluorescence and Wilson and Bacic (2012) for TEM.

### 2.7 Confocal laser‐scanning microscopy analysis

Fluorescence immunolocalization experiments were carried out following the protocol from Coimbra et al. (2007), and imaging was performed on a Leica SP5 microscope (Leica Microsystems, Germany) using laser beam lines of 405 nm (calcofluor white) and 488 nm (FITC; Alexa Fluor 488). Emitted fluorescence was captured between 415 and 455 nm for calcofluor white and between 500 and 550 nm for FITC and Alexa Fluor. Images were analyzed with Zeiss Zen software and Fiji (Schindelin et al., 2012).

### 2.8 Transmission electron microscopy (TEM)

The protocol for preparation of plant cells for TEM was adapted from Wilson and Bacic (2012). The grids were viewed using a FEI Tecnai Spirit transmission electron microscope (FEI, USA) equipped with a GATAN CCD Camera (GATAN Inc., USA). Image analysis was performed with ImageJ (Schneider, Rasband, & Eliceiri, 2012) software (http://rsb.info.nih.gov/ij/) to measure the cell wall thickness and gold density.

### 2.9 Carbohydrate Analysis of cell walls

Approximately 100 mg of material from a 3‐cm segment of the stem base from 4‐month‐old *CALPAIN OE*, wt, and *amiRNA‐DEK1* plants was used to prepare an alcohol‐insoluble residue (AIR) cell wall preparation as described by Pettolino, Walsh, Fincher, and Bacic (2012). Two biological replicates and two technical replicates were used for each line. Samples were analyzed for monosaccharide and linkage composition (acidic and neutral sugars) as described in Pettolino et al. (2012). Crystalline cellulose content of AIR preparations was estimated using the acetic/nitric acid‐based protocol adapted from Updegraff (1969) as described in Pettolino et al. (2012).

### 2.10 Quantification of lignin by the Klason method

Lignin content of the stem (approximately 20 mg) from a 3‐cm segment of the stem base from 4‐month‐old *CALPAIN OE*, wt, and *amiRNA‐DEK1* plants was estimated following the protocol of Theander and Westerlund (1986). Two biological replicates and two technical replicates were used for each line.

## 3 RESULTS

### 3.1 *Overexpression of CALPAIN and reduced expression of* DEK1 *result in altered stem height and diameter*

**Figure 1:**
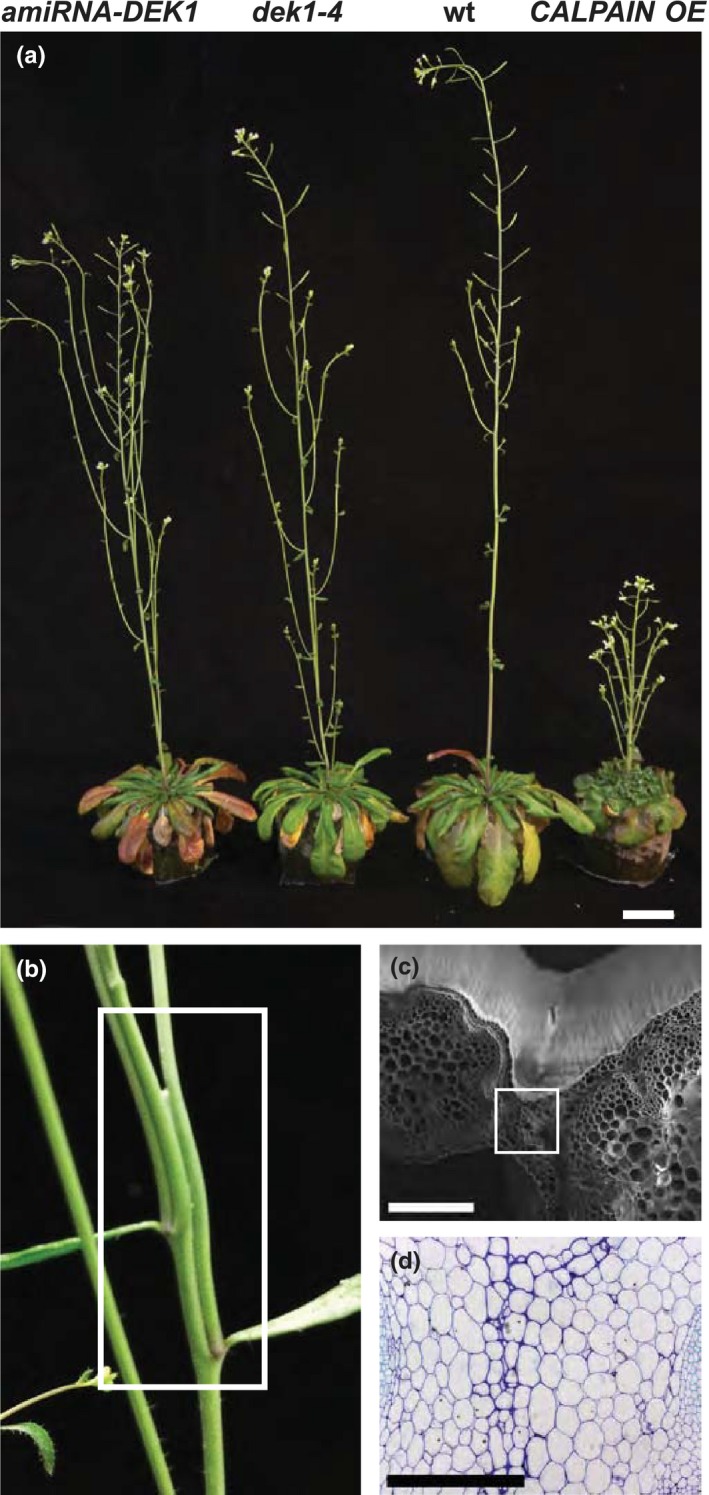
Stem phenotypes of *amiRNA‐DEK1, dek1‐4,* and *CALPAIN OE* plants. (a) Representative picture of 4‐month‐old short‐day‐grown plants showing differences in morphology and stem length. Stem height is decreased in *amiRNA‐DEK1* and *dek1‐4*. *CALPAIN OE* plants show a stunted phenotype (scale bar = 2 cm). (b) Inflorescence stem of long day‐grown *CALPAIN OE* plant showing fusion between main stem and branch at the third and fourth nodes. (c) Cryo‐SEM image of a cross‐sectional fracture of a *CALPAIN OE* stem at the point of stem fusion (scale bar = 200 μm). (d) Light micrograph of a cross section taken at the point of stem fusion as shown by the white box in (c) stained with toluidine blue (scale bar = 250 μm)

Plants overexpressing the active CALPAIN domain of DEK1 (*CALPAIN OE*) in the *dek1‐3* mutant background (Johnson et al., 2008), plants constitutively expressing an artificial microRNA targeting the *DEK1* transcript (*amiRNA‐DEK1*) (Galletti et al., 2015), and the hypomorphic *dek1‐4* allele containing a single missense mutation (Roeder et al., 2012) were used to investigate the effects of DEK1 on inflorescence stem growth. The *dek1‐3* allele is embryo‐lethal suggesting complete loss of DEK1 activity (Johnson et al., 2005; Lid et al. 2005). The overexpression of *CALPAIN* in this background can bypass normal DEK1 regulation (Johnson et al., 2008) and enabled us to observe whether opposite effects occur to *dek1‐4* and *amiRNA‐DEK1* plants. The levels of *CALPAIN* transcript are increased 15‐fold in the *CALPAIN OE* line and a 2.5‐fold reduction in *DEK1* levels occurs in *amiRNA‐DEK1* (Amanda et al., 2016; Galletti et al., 2015; Johnson et al., 2008). We compared inflorescence stem development in short‐day‐grown 4‐month‐old plants, when the stem almost reaches its final height (Boyes et al., 2001), and observed differences between *CALPAIN OE*,* amiRNA‐DEK1,* and *dek1‐4* plants compared to wt (Figure 1a).

Both *dek1‐4* and *amiRNA‐DEK1* plants had shorter and thinner stems compared to wt (Fig. S1). Of these, the *amiRNA‐DEK1* line had the stronger phenotype, as plants displayed a greater height reduction and frequently lodged, bending over at approximately 0.5‐1 cm from the base (Fig. S2). Given the similarity between the *dek1‐4* and *amiRNA‐DEK1* phenotypes and the more obvious phenotypes of the latter, the *amiRNA‐DEK1* line was used for further analysis. The height of *amiRNA‐DEK1* plants was reduced by approximately 60% whereas the stem diameter at the base was reduced by approximately 7% compared to wt (Fig. S1; Table 1). No obvious phenotypic differences in the final length of lateral branches of *amiRNA‐DEK1* plants were observed (Figure 1a). Given the prostrate phenotype, we investigated whether stem biomechanical properties were altered in DEK1 transgenic lines using a three‐point bending test. Flexural strength is defined as the maximum stress a sample withstands before it yields/breaks, and stiffness is a measure of the modulus of elasticity of the stem or the force required to deform the sample over a unit distance; the cross‐sectional diameter of the sample is included in each calculation (MacMillan et al., 2010). Tests were performed using the basal 6‐cm segment of the stem for wt and *amiRNA‐DEK1* and a 3‐cm segment for *CALPAIN OE* due to the reduced plant height (Figure 1a). A statistically significant reduction in flexural strength was observed in the basal segment of *CALPAIN OE* plants. No statistically significant difference in flexural stiffness was observed in either *amiRNA‐DEK1* or *CALPAIN OE* plants compared to wt (Table 1).

**Table 1:** Stem morphology measurements of 4‐month‐old short day‐grown calpain oe, wt, and amiRNA‐DEK1 plants^a^

	*CALPAIN OE*	wt	*amiRNA‐DEK1*	n^b^
Gross morphology of stem				
Height (cm)	**2.3 (± 0.1)** c	34.1 (± 0.3)	**18.8 (± 0.4)**	15
Diameter of base (mm)	**1.38 (± 0.02)**	0.95 (± 0.01)	**0.88 (± 0.01)**	10
Diameter at ≥3 cm (mm)	**1.27 (± 0.02)**	2.07 (± 0.06)	1.79 (± 0.02)	8‐10
Diameter‐to‐height ratio	**6.53 (± 0.82)**	0.25 (± 0.01)	**0.5 (± 0.01)**	10
Stem biomechanical properties				
Flexural stiffness (Mpa)	2208.2 (± 307.3)	2846.2 (± 133.3)	2446.2 (± 158.0)	9
Flexural strength (Mpa)	**30.9 (± 2.5)**	42.4 (± 4.3)	33.7 (± 3.6)	9
Tissue and cell morphology				
Cortex cell numbers/0.05 μm^2^	**17.2 (± 0.3)**	22.6 (± 0.4)	**27.7 (± 0.4)**	5
Numbers of cortex layer	7	4‐5	4‐5	5
Tissue layer width (μm):				
Epidermis	**35.4 (± 0.4)**	30.0 (± 0.2)	28.2 (± 0.1)	35
Cortex	**372.7 (± 1.4)**	231.4 (± 1.2)	235.4 (± 1.1)	20‐25
IFR	**121.1 (± 1.1)**	133.8 (± 0.7)	**170.2 (± 0.8)**	20‐25
Pith	**388.7 (21.5)**	219.4 (± 12.2)	106.8 (± 7.9)	10
Cell size (μm^2^):				
Epidermis	**2233.8 (± 53.6)**	1362.2 (± 31.4)	945.8 (± 15.4)	20
Cortex	**5093.4 (± 32.9)**	2690.3 (± 27.1)	**966.9 (± 7.5)**	30
IFR	**1725.9 (± 25.8)**	858.1 (± 17.9)	828.4 (± 21.4)	20
Pith	**14655.1 (± 93.6)**	6337.7 (± 79.2)	**2217.5 (± 28.9)**	30
Cell wall thickness (μm)^d^				
Epidermis	**2.07**	0.95	**1.71**	10
Cortex C1 outer periclinal	**0.22**	0.12	**0.10**	10
Cortex C1 anticlinal	**0.06**	0.03	0.03	10
Cortex C1 inner periclinal	**0.10**	0.06	**0.04**	10
Cortex C2	0.03	0.03	0.03	10
IFR	**0.91**	2.77	**0.55**	10
Xylem	0.77	0.77	0.73	10
Phloem	**0.24**	0.16	**0.14**	10
Pith	0.28	0.29	**0.22**	10

a Bold text indicates statistically significant value at *p* < .05 using oneway ANOVA.

b For stem and tissue morphology and biomechanical properties, n represents biological replicates. For cell size, n represents number of cells taken from 2 biological replicates for each line. For cell wall thickness measurements, *n* represents the number of cells from 2 biological replicates. For each cell 5 areas of the cell wall were measured.

c All values shown in brackets are standard error.

d *SE* < 0.01 each case, therefore are not reported.

In contrast to plants with reduced DEK1 activity and wt plants, *CALPAIN OE* plants exhibit a stunted phenotype with an average stem height of 2.3 cm (Figure 1a; Table 1; Fig. S2). Node lengths were also dramatically reduced in length giving the plants a “bushy” appearance (Figure 1a; Fig. S2). In addition, *CALPAIN OE* plants often showed fasciation (stem flattening) and fusion of lateral branches to the main stem. Fusions are more severe in long day‐grown plants where the stem length was longer (Figure. 1b‐d). The diameter at the basal part of *CALPAIN OE* stems is larger than that of wt, whereas at 3 cm above the base the main stem is thinner than that of wt, and often difficult to distinguish from branches due to fusion (Table 1). Where primary and secondary branches are fused, the epidermal layers are difficult to discern (Figure 1d).

### 3.2 Altered levels of DEK1 activity cause changes in cellular morphology of the stem

In addition to DEK1 having a role in epidermal development (Becraft et al., 2002; Galletti et al., 2015; Hibara et al., 2009; Johnson & Ingram, 2005; Johnson et al., 2008; Lid et al., 2005; Roeder et al., 2012), *dek1* mutants also display defects in other cell types (Ahn et al., 2004; Perroud et al., 2014). Thus, we investigated cellular morphology and ultrastructure of all cell types in the stem of 4‐month‐old short‐day‐grown plants using light and transmission electron microscopy (TEM).

Due to the differences in stem height and morphology, exemplified in the stem diameter‐to‐height ratio (Table 1), determining equivalent developmental stages among the different genotypes was problematic. Hence, we decided to carry out tissue morphological studies at 2 mm above the stem–root junction, as this was likely to be the most developmentally comparable region.

**Figure 2:**
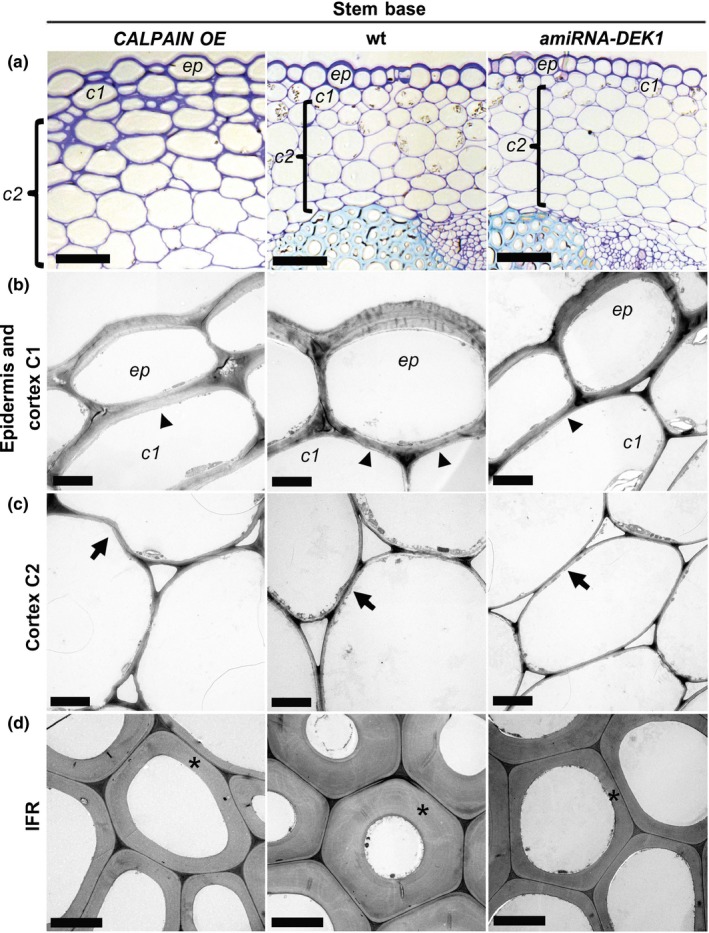
Light and electron micrographs demonstrating the morphology of epidermis, cortex, and interfascicular fiber region (IFR) cells in the basal part of 4‐month‐old *CALPAIN OE*, wt, and *amiRNA‐DEK1* stems. (a) Cross sections at the stem base stained with toluidine blue show differences in cellular morphology and increased width of the cortex layer in *CALPAIN OE* (scale bar = 100 μm). (b) to (d) Representative TEM transverse sections of cells in the epidermal layer and cortex C1 (b), cortex C2 (c), and IFR (d). *CALPAIN OE* shows increased cortex C1 layer wall thickness (arrowhead) and changes in cortex C2 layer wall shape (arrow), and both *CALPAIN OE* and *amiRNA‐DEK1* show reduced secondary cell wall thickness (asterisk) in interfascicular fiber region (IFR) cells (d) (scale bar = 5 μm). (ep = epidermis; c1 = cortex cell layer 1; c2 = cortex cell layers 2)

In cross sections obtained from the base of the stem of wt and *amiRNA‐DEK1* plants, the epidermis forms a single layer of cells with uniform size and a thickened outer periclinal wall (Figure 2a). In contrast, epidermal cells in *CALPAIN OE* stems are less uniform in size and are disorganized (Figure 2a). In addition, the width of tissue layers in the epidermis, cortex, and pith regions is all expanded in *CALPAIN OE* plants, a factor that presumably contributes to the increased stem diameter (Table 1). The size of cells within these layers was quantified, and in *CALPAIN OE,* the epidermis, cortex, and pith cell size was found to be increased compared to wt (Figure 2a; Fig. S3; Table 1). In addition to the increased cell size, the number of cortical cell layers observed in *CALPAIN OE* plants was also increased, resulting in a greater contribution of the cortex region to stem diameter (Table 1; Figure 2a; Fig. S3).

The *CALPAIN OE* cortex can be differentiated into two types of cell layers based on cellular structure: C1, a single cell layer directly adjacent to the epidermis, and C2, the remaining cell layers in the cortex (Figure. 2a‐c). The C1 layer of *CALPAIN OE* consists of cells of variable size (Figure 2b) and has thickened cell walls like those of the epidermis. In the cortex C2 layers of *CALPAIN OE* stems, the shape of cells is also irregular compared to wt (Figure 2a, c). Cross sections of the stem in wt reveal rounded cells with smooth walls, whereas in *CALPAIN OE* plants, cells have a slightly flattened appearance with kinked walls suggesting altered cell–cell contacts in *CALPAIN OE* (Figure 2a, c). Defects in walls were also apparent in pith cells of the *CALPAIN OE* lines (Fig. S4). No obvious differences were observed in the vascular tissues of *CALPAIN OE* plants compared to wt (Fig. S4 b‐d). As cell shape is influenced by the properties of the cell wall, this phenotype might reflect localized changes in composition, organization, and/or thickness of the cell wall.

In wt stem sections, a thickened epidermal cell wall, as compared to the thinner cortical cell walls, is clearly visible (Figure 2b, c). The epidermal cell walls of both *CALPAIN OE* and *amiRNA‐DEK1* plants showed an increased thickness compared to the wt (Table 1). In addition, *CALPAIN OE* plants show increased thickening of the walls in the cortical C1 layer (Figure 2b; Table 1). Specifically, the inner and outer periclinal (adjacent wall to the epidermal cell) and anticlinal (between C1 cortical cells) walls were thicker compared to wt and *amiRNA‐DEK1* (Table 1). In contrast, *amiRNA‐DEK1* plants show a decrease in the thickness of outer and inner periclinal C1 walls (Table 1). The thickness of cell walls in the C2 layer of cortical cells is similar in all the lines studied (Figure 2c; Table 1). Finally, increased and decreased wall thickness was observed in the phloem cells of *CALPAIN OE* and *amiRNA‐DEK1* lines, respectively (Table 1).

An approximately 7% decrease in stem diameter is seen in *amiRNA‐DEK1* plants, with a significant decrease in cell size observed in the cortex and pith layers with no obvious differences in cell shape (Figure. 2a; Figs. S3, S4). The cells that appear to be most affected in *amiRNA‐DEK1* stems are phloem, xylary procambium cells in vascular bundles, and interfascicular fiber region (IFR) cells (Fig. S4). Phloem and xylary procambium cells sometimes appeared collapsed and this is likely to contribute to the weak stem phenotype (Fig. S4b, d). Interestingly, an increase in the width of the IFR layer was observed in *amiRNA‐DEK1* lines, with no obvious difference in cell size, suggesting the presence of an additional IFR cell layer (Table 1). In contrast to *amiRNA‐DEK1*, a decrease in the width of the IFR region is observed in *CALPAIN OE* lines compared to wt, associated with increased cell size, indicating the presence of fewer cell layers (Table 1). In addition to the changes in IFR cell number, we observed that cell wall thickness in the IFR of both *CALPAIN OE* and *amiRNA‐DEK1* stems was significantly reduced compared to wt (Figure 2; Table 1; Fig. S4a). Taken together, these results suggest that changes in stem diameter in *CALPAIN OE* and *amiRNA‐DEK1* lines are due to changes in cell number and size, most notably in the cortex, IFR, and pith layers.

### 3.3 Altered DEK1 levels affect cell wall composition

As the size and shape of cells are largely determined by the structure and composition of the cell walls, we investigated whether the altered cellular phenotypes observed in *CALPAIN OE* and *amiRNA‐DEK1* occur because of changes in cell wall morphology, composition, and/or deposition.

As lignin is a major polymer of the IFR cell wall (Zhong, Ripperger, & Ye, 2000), we measured Klason lignin in a 3‐cm basal region of 4‐month‐old stems. Although a reduction in lignin content was detected in both *CALPAIN OE* and *amiRNA‐DEK1*, the difference was not significant (Table 2).

**Table 2:** Average gold density measurement/0.1 μm^2^ walls for selected cell wall epitopes in 4 month‐old stem base and cell wall content analysis of stems^a^^,^ b

	*CALPAIN OE*	wt	*amiRNA‐DEK1*	n^c^
Cellulose (CBM3a):				
Epidermis	**13.0 (± 0.6)** d	7.5 (± 0.9)	4.8 (± 0.3)	5
Cortex C1	3.7 (± 0.9)	3.9 (± 0.5)	2.7 (± 0.1)	5
Cortex C2	**8.1 (± 0.4)**	2.4 (± 0.3)	3.1 (± 0.1)	5
Xylem	**12.7 (± 0.4)**	8.1 (± 0.4)	**2.1 (± 0.2)**	5
IFR	**13.2 (± 0.6)**	2.4 (± 0.1)	1.5 (± 0.1)	5
Low DE pectin (JIM5):				
Cortex C1	17.9 (± 0.5)	20 (± 0.7)	18 (± 0.5)	5
Cortex C2	**39.2 (± 1.8)**	26.1 (± 1.0)	34.7 (± 0.9)	5
IFR	**36.9 (± 1.9)**	10.8 (± 0.7)	7.9 (± 0.7)	5
High DE pectin (JIM7):				
Cortex C1	5.4 (± 0.4)	5.1 (± 0.2)	5.1 (± 0.1)	5
Cortex C2	**36.7 (± 1.9)**	5.7 (± 0.2)	7.4 (± 0.6)	5
IFR	**7.0 (± 0.4)**	3.9 (± 0.2)	4.2 (± 0.3)	5
Cell wall content (%):				
Klason lignin	17.6 (± 1.3)	21.4	17.6 (± 0.9)	2
Pectin	38.9	33.6	33.0	2
Crystalline cellulose	47.3	48.0	46.3	2

a Bold text indicates statistically significant value at *p* < .05 using oneway ANOVA.

b A 3 cm segment of the basal stem region was used for cell wall analysis.

c For antibody labelling, n represents 5 cells from 2 biological replicates. For cell wall analysis *n* is biological replicates.

d All values shown in brackets are standard error, *SE* < 0.1 are not reported.

To determine whether altering DEK1 activity causes changes in total wall carbohydrate composition, monosaccharide and polysaccharide compositional analyses of an alcohol‐insoluble residue (AIR; Pettolino et al., 2012) from a 3‐cm basal region of the stem of the 4‐month‐old basal inflorescence of *CALPAIN OE*, wt, and *amiRNA‐DEK1* were undertaken. Monosaccharide and linkage analyses indicated minor changes in cellulose, pectins, type I and type II arabinogalactans, and glucuronoxylan (Fig. S5). Further assays to measure pectins and crystalline cellulose (Updegraff, 1969) were undertaken due to their involvement in stem mechanics. Whereas increased levels of pectins were detected in *CALPAIN OE* stems, there were no changes in crystalline cellulose content (Table 2). No significant differences in pectins or crystalline cellulose were detected in *amiRNA‐DEK1* stems. To gain more information about potential subcellular changes that might not be detected in analyses of total stem samples, we used immunolocalization with antibodies that recognize specific cell wall polysaccharides.

### 3.4 Cellulose and pectin content in the primary walls are affected by DEK1

Initial immunofluorescence studies were undertaken using a range of antibodies directed against epitopes of cell wall polysaccharides identified in the linkage analysis, including HG, galactan, arabinan, and xyloglucan (XG) to determine the labeling patterns (Fig. S6). Differences in the intensity of labeling were only observed for HG pectins (Fig. S6) and this was investigated further. Pretreatment (demasking) with xyloglucanase was performed to ensure accessibility of the antibodies as XGs are known to mask labeling of pectins (Marcus et al., 2008).

**Figure 3:**
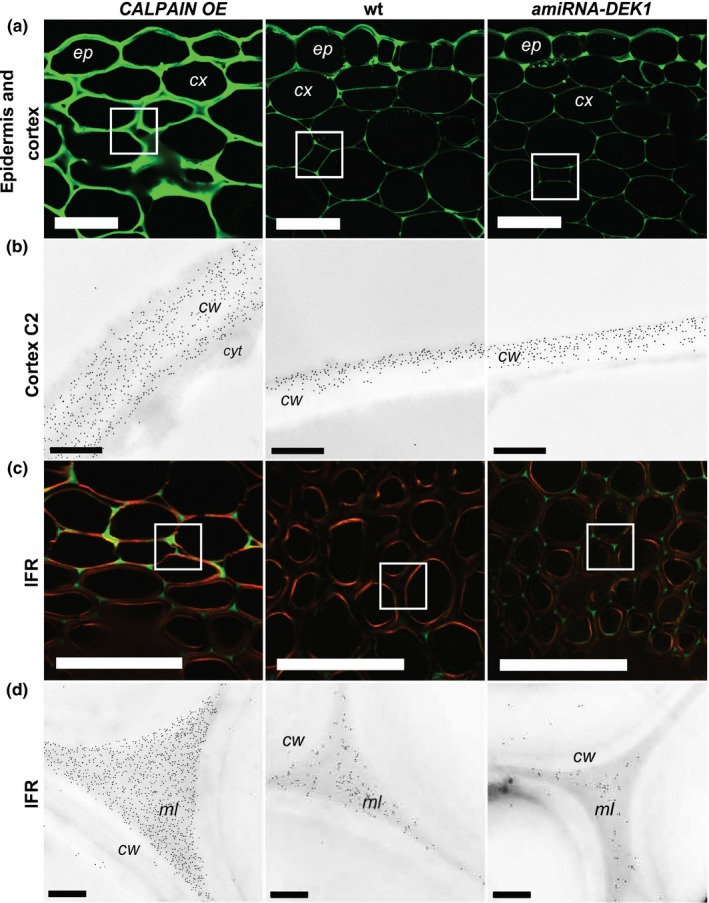
Detection of JIM5 (low DE pectin) epitopes in cross sections of the basal part of 4‐month‐old *CALPAIN OE*, wt, and *amiRNA‐DEK1* stems after xyloglucanase pretreatment. (a) Immunofluorescence labeling shows higher intensity in epidermis and cortex cells walls in *CALPAIN OE* compared to wt and *amiRNA‐DEK1* (scale bar = 25 μm). (b) TEM immunogold labeling in the cortex C2 cell walls (white box in A) shows increased labeling in *CALPAIN OE* walls (scale bar = 0.5 μm). (c) Immunofluorescence labeling of IFR cells shows higher intensity in the middle lamella of *CALPAIN OE* (scale bar = 25 μm). (d) TEM immunogold labeling of middle lamella of IFR cells shows increased and decreased gold labeling in *CALPAIN OE* and *amiRNA‐DEK1*, respectively (scale bar = 0.5 μm). (cw = cell wall; cyt = cytoplasm; ml = middle lamella; ep = epidermis; cx = cortex)

**Figure 4:**
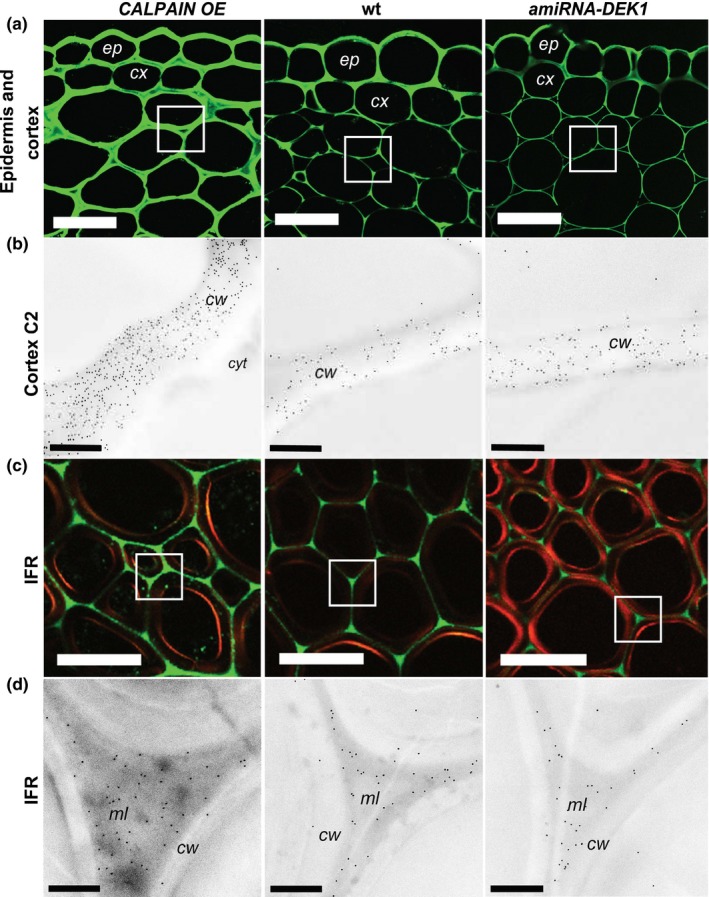
Detection of JIM7 (high DE pectin) epitopes in cross sections of the basal part of 4‐month‐old *CALPAIN OE*, wt, and *amiRNA‐DEK1* the stems after xyloglucanase treatment. (a) Immunofluorescence labeling shows higher intensity in epidermis and cortical cell walls in *CALPAIN OE* compared to wt and *amiRNA‐DEK1* (scale bar = 25 μm). (b) TEM immunogold labeling in the cortex C2 layer cell walls (white box in (a)) shows increased labeling in *CALPAIN OE* walls (scale bar = 0.5 μm). (c) Immunofluorescence labeling of IFR cells shows higher intensity in the middle lamella of *CALPAIN OE* (scale bar = 25 μm). (d) TEM immunogold labeling of middle lamella of IFR cells shows increased gold labeling in *CALPAIN OE* (scale bar = 0.5 μm). (cw = wall; cyt = cytoplasm; ml = middle lamella; ep = epidermis; cx = cortex)

A clear difference in the intensity of labeling between the different lines was evident with the anti‐HG pectin antibodies JIM5, which recognizes HG with a low degree of methylesterification (DE) and JIM7, which recognizes HG with a high DE (Knox et al., 1990) in the cortex and IFR cells. Sections from *CALPAIN OE* plants showed significantly higher fluorescence intensity with the JIM5 antibody compared to wt and *amiRNA‐DEK1* (Figure 3). The labeling pattern in *CALPAIN OE* was also different to that of wt and *amiRNA‐DEK1*, with cortical cell walls being uniformly labeled, rather than mainly labeled at the junctions between cells as observed in wt (Figure 3a). In addition, *CALPAIN OE* sections showed stronger fluorescence in the junctions between IFR cells than wt (Figure 3c). Labeling with the JIM7 antibody (Figure 4a, c) shows a similar pattern to that of JIM5, with labeling being more abundant in *CALPAIN OE* cortical cells and in the middle lamella between IFR cells.

TEM was used to examine the distribution of JIM5 and JIM7 epitopes within individual cell walls using immunogold labeling. *CALPAIN OE* stem sections showed significantly increased gold density in cortex C2 layer cell walls and the middle lamella between IFR cells with both JIM5 (Figure 3b,d) and JIM7 (Figure 4b,d) labeling (Table 2), consistent with immunofluorescence results. The distribution of JIM5 labeling in the cell wall of cortex C2 cells differs in *CALPAIN OE* compared to wt. Whereas in wt, and to a lesser degree *amiRNA‐DEK1*, JIM5 labeling is localized to the cell wall edges, in *CALPAIN OE* it is uniformly distributed throughout the wall (Figure 3b). No difference in immunogold labeling was observed in the epidermal and cortex C1 layers (Fig. S7; Table 2). Furthermore, no significant difference in immunogold labeling was detected between *amiRNA‐DEK1* and wt stems (Table 2).

**Figure 5:**
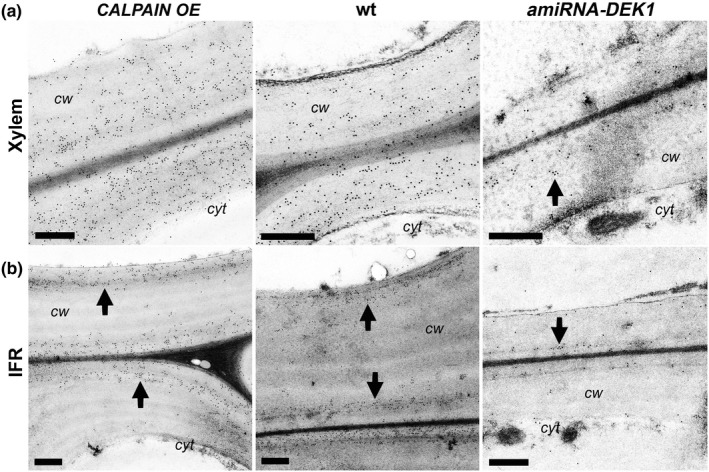
TEM immunogold labeling of cellulose epitopes by CBM3a in the stem base of *CALPAIN OE* and *amiRNA‐DEK1* plants in cells with secondary cell wall thickening. Increased gold labeling is seen in xylem (a), and IFR (b) walls of *CALPAIN OE*, while the opposite is seen in xylem walls of *amiRNA‐DEK1*. (Arrows indicate the location of gold particles; cw = cell wall; cyt = cytoplasm; scale bar = 0.5 μm)

We also investigated crystalline cellulose using the crystalline cellulose‐directed CBM3a protein (Blake et al., 2006) as cellulose is also known to influence the biomechanical properties of stems. TEM shows an increase of CBM3a labeling in IFR, xylem, epidermal, and cortex C1 cell walls of *CALPAIN OE* stems compared to wt (Figure 5; Fig. S8; Table 2). A significant reduction in CBM3a labeling was only observed in the cell walls of the xylem of *amiRNA‐DEK1* with no difference in the cell walls of other cell types (Figure 5; Table 2).

## 4 DISCUSSION

Previous studies of plants with reduced DEK1 activity support a role for DEK1 in the regulation of epidermal development in cotyledons, leaves, and sepals (Galletti et al., 2015; Johnson et al., 2008; Roeder et al., 2012). Here, we provide evidence that DEK1 is also required for normal development of the inflorescence stem. Analysis of cell wall composition and ultrastructure suggests that DEK1 has a role in regulating the deposition of cellulose and pectin and that these changes likely result in altered mechanical properties in primary and secondary walls leading to defects in stem development.

Growth of the stem involves a complex balance between cell elongation and strength maintenance/reinforcement. This in turn requires tight coordination between cells and tissues with vastly different developmental programs and wall types (primary and secondary). Importantly, the deposition of rigidifying secondary cell walls, required for mechanical support, must be temporally coordinated with cell elongation, necessary for axial growth. The importance of balanced cell proliferation and expansion in the epidermis and underlying layers during stem development is elegantly demonstrated from studies of double mutants in *CLAVATA3 and DE‐ETIOLATED* (*clv3‐8 det3‐1*) that show cracks in the stem (Maeda et al., 2014). This is proposed to occur due to an increased inner stem volume as a result of the *clv3‐8* mutation that exerts an outward mechanical stress. In the *det3‐1* background, which is defective in cell expansion, this results in cracking of the epidermal cell layer.

Roles for DEK1 in maintenance of the epidermal layer and regulation of growth coordination have previously been shown in the leaf (Johnson et al., 2008). Our results suggest that DEK1 is involved in regulating cell wall synthesis and remodeling to ensure the coordination of cell wall deposition with other developmental cues. This is supported by transcriptomic studies of *Arabidopsis* plants overexpressing the CALPAIN domain (Johnson et al., 2008) and *Physcomitrella* lacking the *dek1* gene (Demko et al., 2014). Among the genes whose expression is significantly misregulated in these studies, cell wall‐related genes are overrepresented compared to wt controls. More recently, the expression of several genes involved in the biosynthesis and remodeling of the cell wall has been correlated with increased *CALPAIN* expression (Amanda et al., 2016). These transcriptional changes in response to changes in DEK1 activity relate to changes in the levels of pectin and cellulose in the leaf epidermis. Based on the observed stem phenotypes reported here, we propose that changes in the cell wall deposition and/or organization in plants with altered DEK1 activity also impact the coordination of growth within and between cell layers in stems, resulting in altered stem architecture.

In *CALPAIN OE* plants, pectin levels are increased, the deposition of both pectin and cellulose is altered and significant changes in cell wall thickness are observed. Given the role of pectin and cellulose in the maintenance of tensile strength in the cell wall, it seems likely that a combination of changes in these polymers could be the cause of altered stem growth. Recent studies suggest that pectins make the majority of contacts with cellulose and act as mechanical tethers between cellulose microfibrils (Wang et al., 2012). Peaucelle, Wightman, and Hofte (2015) showed that pectin demethylesterification influences the re‐orientation of cellulose to enable cell elongation in *Arabidopsis* hypocotyls. Altered stem height and width observed in *CALPAIN OE* stems might be caused by changes in cellulose microfibril orientation and strengthening of the cellulose–pectin network. Cell wall thickness is also increased in nearly all cell types of the stem in *CALPAIN OE* plants and this could either be a cause or a consequence of reduced elongation. Investigation of cell wall thickness during earlier stages of stem growth will be required in future studies to distinguish causes from effects.

How changes in DEK1 activity influence cellulose synthesis and deposition is of major interest. Cellulose is synthesized by *CELLULOSE SYNTHASES* (*CESAs*) that form cellulose synthase complexes (CSCs) (rosettes) located at the plasma membrane (McFarlane, Döring, & Persson, 2014). Disruption of CESAs involved in making cellulose in secondary cell walls results in reduced cellulose content and secondary wall thickening in rice, *Brachypodium*, and *Populus* (Handakumbura et al., 2013; Joshi et al., 2011; Tanaka et al., 2003). Mutants with reduced levels of cellulose include a number of *IRREGULAR XYLEM* (*irx*) mutants that show collapsed xylem and reduced secondary wall thickness (Turner & Somerville, 1997). The importance of pectins for stem strength is also demonstrated by the *irx8* mutant defective in the expression of *GAUT12*, which encodes a putative glycosyltransferase involved in HG synthesis. *irx8/gaut12* mutants show a severely dwarfed phenotype, collapsed xylem, and reduced xylan, cellulose, pectin, and lignin deposition (Hao et al., 2014; Pena et al., 2007; Persson et al., 2007). In the apoplast, HG can be demethylesterified by pectin methylesterases (PMEs) that are regulated by PME inhibitors (PMEIs) (Di Matteo et al., 2005; Giovane et al., 2004; Juge, 2006; Micheli, 2001). The importance of appropriate methylesterification of HG is shown by phenotypes in mutants with altered activity of either PMEs or PMEIs. For example, in a *pme35* mutant, increased levels of high DE HG in the cell walls of cortical and IFR cells were shown to influence stem mechanical properties resulting in a prostrate stem phenotype (Hongo et al., 2012). The organ fusions observed in *CALPAIN OE* could also be a result of changes in pectins and/or epicuticular waxes (Peaucelle et al., 2011; Weng, Molina, Shockey, & Browse, 2010). Investigation of the expression of genes encoding CESAs, PMEs, PMEIs, and pectin biosynthetic enzymes in *CALPAIN OE* and *amiRNA‐DEK1* plants during stem growth is thus a logical future extension of this current work that will aid our understanding of how DEK1 activity regulates cellulose and pectin levels.

The stem phenotypes in *CALPAIN OE* plants are likely to be associated with constitutive activity of the CALPAIN domain leading to misregulation of cell wall‐related gene expression and altered cell wall composition/organization. A question remains as to how this fits with previously described roles for DEK1 in specification and maintenance of the epidermis. Epidermal cell walls play a crucial role in counteracting the outward mechanical forces imposed by internal tissues during stem growth. We propose that one possible function of DEK1 is to control epidermal cell walls in order to maintain tissue integrity. Our results in the stem support a role for DEK1 in coordinating growth between the epidermis and underlying cortex cells. This is consistent with previously observed *CALPAIN OE* phenotypes in the leaf, where additional mesophyll cell layers occur (Johnson et al., 2008). The cortex has been reported to contribute to the mechanical support of the stem, and the thickness of this layer is shown to increase after mechanical perturbation (Paul‐Victor & Rowe, 2010). We propose that an increased number of cortex cell layers could impart mechanical support to the *CALPAIN OE* stem. Additionally, increased cell wall thickness in the cortex C1 layer could restrict the effects of outward mechanical forces. Three‐point flexural tests of *CALPAIN OE* stems show reduced flexural strength. Changes in stem diameter and cell wall thickness in *CALPAIN OE* plant likely provide stiffness levels similar to wt, whereas the reduced thickness of secondary cell walls in IFR could contribute to reduced flexural strength given the importance of lignin for providing compressive and bending strength (MacKay et al., 1997). In future, tensile tests would be beneficial to investigate further the biomechanical properties of *CALPAIN OE* plants. Interestingly, cortical C2 layer cells in *CALPAIN OE* plants also display altered cell–cell contacts resulting in regions where the walls are kinked. In epidermal pavement cells, a jigsaw‐like shape occurs when cells expand due to the differences in wall thickening and tight cell–cell adhesion. This is believed to enhance the structural integrity of the organ (Ambrose, DeBono, & Wasteneys, 2013). In the *CALPAIN OE* line, kinked walls could be caused by either uneven thickening of the cell walls, localized differences in cell wall composition in these cells, or uncoordinated growth between neighboring cells (Higaki et al., 2016); however, this requires further investigation.

The epidermis and cuticle also play an important role as the boundary with the environment. Defects in the cuticle are likely to contribute to the observed fusions between the branches and main stem of *CALPAIN OE* plants, as the cuticle acts as the primary barrier preventing organ fusion (Javelle et al., 2010; Takada, Takada, & Yoshida, 2013; Wu et al., 2011). Alternatively, changes in the shape and size of the shoot apical meristem (SAM) or boundary regions could explain the fusion and stem flattening, and this warrants further investigation (Landrein, Kiss et al., 2015; Landrein, Refahi et al., 2015; Liang et al., 2015).

Although no major changes in overall cell wall composition of the stem were observed in *amiRNA‐DEK1* lines, some specific tissue types showed reduced cell wall thickness and alterations in cellulose and pectin levels/distribution. Reduced labeling of crystalline cellulose epitopes in the xylem and reduced cell wall thickness in phloem cells (Tables 1, 2) could lead to the observed collapse of these cells. We propose that the collapse of the xylem procambium and phloem cells may impede the movement of water and nutrients to the apical meristem and this could result in the observed decrease in growth in these lines. An increase in overall cellulose levels with no change in crystalline cellulose suggests *amiRNA‐DEK1* lines may have more amorphous cellulose. Together with the smaller size of cells and reduced stem diameter, this could contribute to the bent *amiRNA‐DEK1* stem phenotype. Future studies analyzing growth kinetics of the stem will provide further insight into how these phenotypes come about.

In this study, a reduction in the size of pith and cortex cells was observed in *amiRNA‐DEK1* stems. Similarly, a reduction in cell size was observed in tobacco plants with reduced *NbDEK1* expression using *virus‐induced gene silencing* (VIGS) where an increased number of smaller cells was seen in every tissue layer of the stem (Ahn et al., 2004). Hyperproliferating cell masses in the epidermis were also observed on *NbDEK1* VIGS stems, and in *Arabidopsis*, callus‐like outgrowths on stems were seen in plants with reduced DEK1 activity (Johnson et al., 2008). These phenotypes are proposed to be due to loss of integrity of the epidermal layer, allowing outgrowth of underlying tissue. However, these phenotypes were not seen in our studies, likely due to the reduction in DEK1 activity in *amiRNA‐DEK1* lines being weaker than in the studies mentioned above.

Future investigation of the timing of cell wall deposition during growth is required to determine how DEK1‐mediated regulation of cellulose and pectin synthesis and deposition in the cell wall leads to the stem phenotypes described in this work. One possibility is that secondary cell wall modifications in at least some cell types are mistimed in our transgenic lines, leading to either inappropriately early growth cessation, or lack of appropriate cell wall modification. Kinetic analysis of cell wall changes will allow us to distinguish the timing of onset of the various changes that we have documented and clarify our view of the direct and more indirect effects of changing DEK1 activity in stems. The contribution of cell wall mechanics in regulating growth and development has gained much attention in recent years, yet little is known about these processes in stems. Future work investigating the role of DEK1 in both stems, and other tissues, will contribute to our knowledge in this important area.

## AUTHOR CONTRIBUTIONS

DA performed the experiments, processed, analyzed, and interpreted the data, and wrote the article with the co‐authors. CPM performed biomechanical tests, processed and interpreted the data, and provided feedback on the article. MSD, AB, and JFG assisted with supervision of DA and with the interpretation of the data and writing of the article. RG and GCI generated and provided amiRNA‐DEK1 and *dek1‐4* in Col plant lines for the experiments and provided scientific advice and assistance with writing of the article. KLJ conceived the original research plan, supervised DA, assisted with the design and interpretation of the data, and wrote the article with DA.
