# Supplementary material for: Arabidopsis DEFECTIVE KERNEL1 regulates cell wall composition and axial growth in the inflorescence stem
Source: Plant Direct. 2017 Dec 6;1(6):e00027. doi: 10.1002/pld3.27 (PMC6508578; doi:10.1002/pld3.27)
Supplement: Supplementary file 1 [file PLD3-1-e00027-s001.pdf]

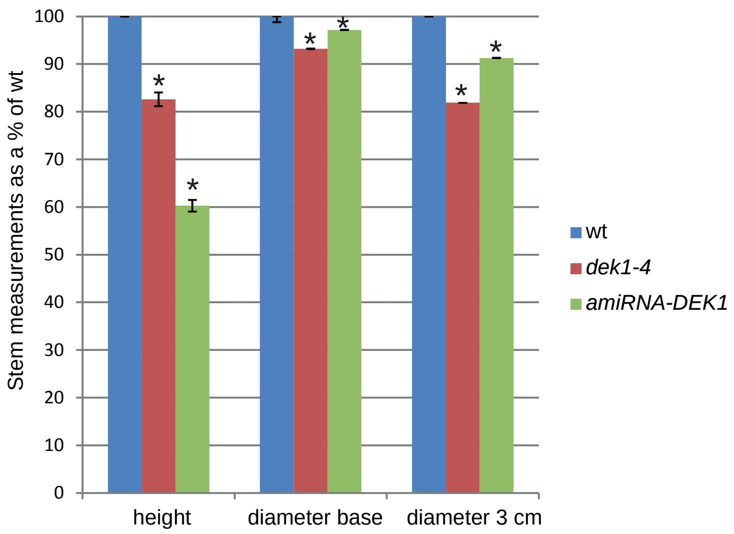

Supplemental Fig. S1. Measurements of stem height and diameter in 4 month-old short day-grown wt, *dek1-4* and *amiRNA-DEK1* plants. Measurements are shown as a percentage of wt and locations outlined in Supplemental Fig. S2. Asterisk denotes a statistically significant value at $p<0.05$ using one-way ANOVA. Error bars show standard error. n = 8 to18

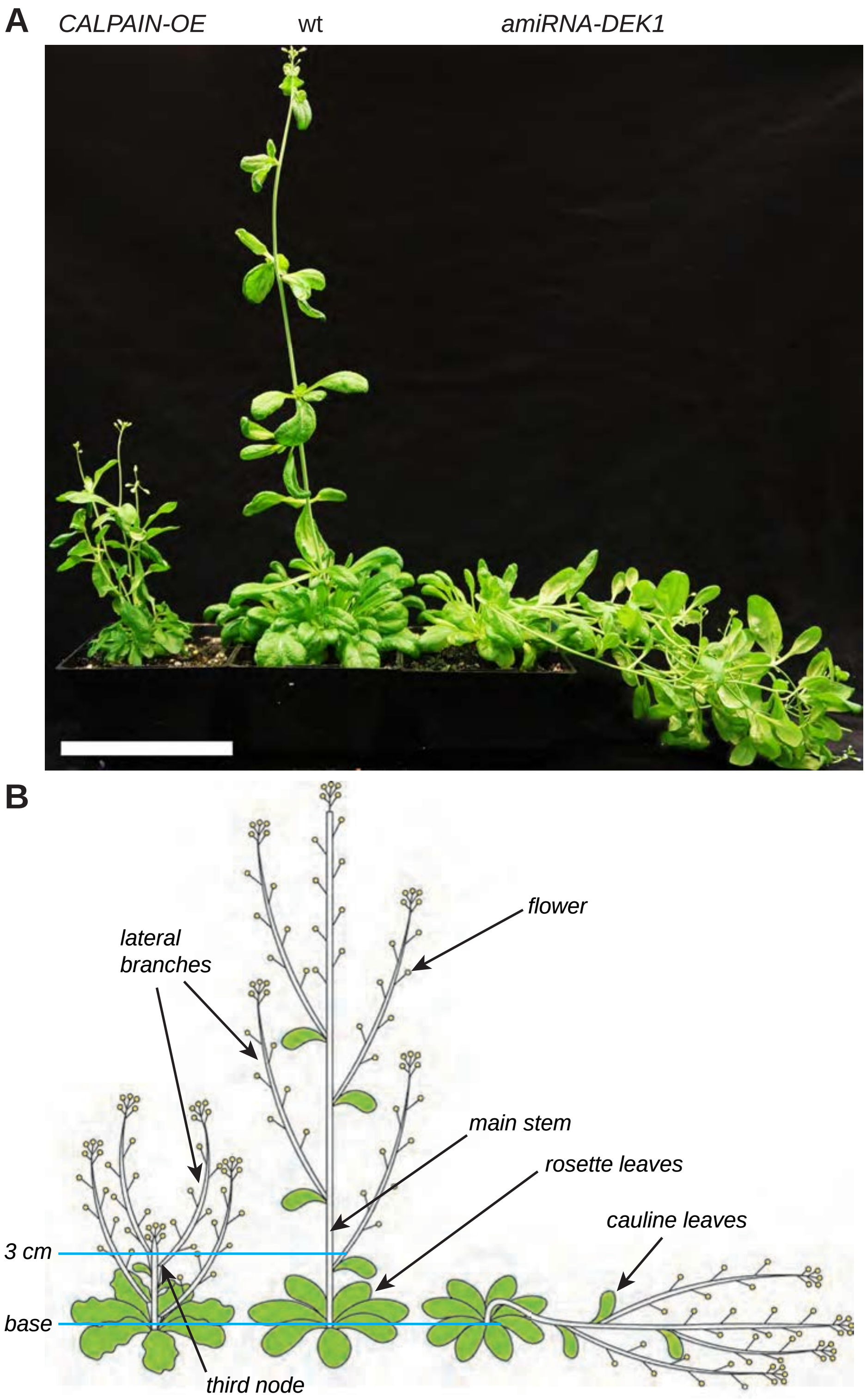

Supplemental Fig. S2. Stem phenotypes and schematic representation of *CALPAIN OE* and *amiRNA-DEK1* plants compared to wt. **(A)** Representative picture of 4 month-old short day-grown plants showing severly decreased height in *CALPAIN OE* plants and prostrate stem phenotype in *amiRNA-DEK1* plants (scale bar = 10 cm). **(B)** Positions where cross sections were taken for histological studies (base and 3 cm from base) are shown by a light blue line. The entire 3 cm section of *CALPAIN OE* stems and the bottom 6 cm of wt and *amiRNA-DEK1* were used for stem biomechanical tests.

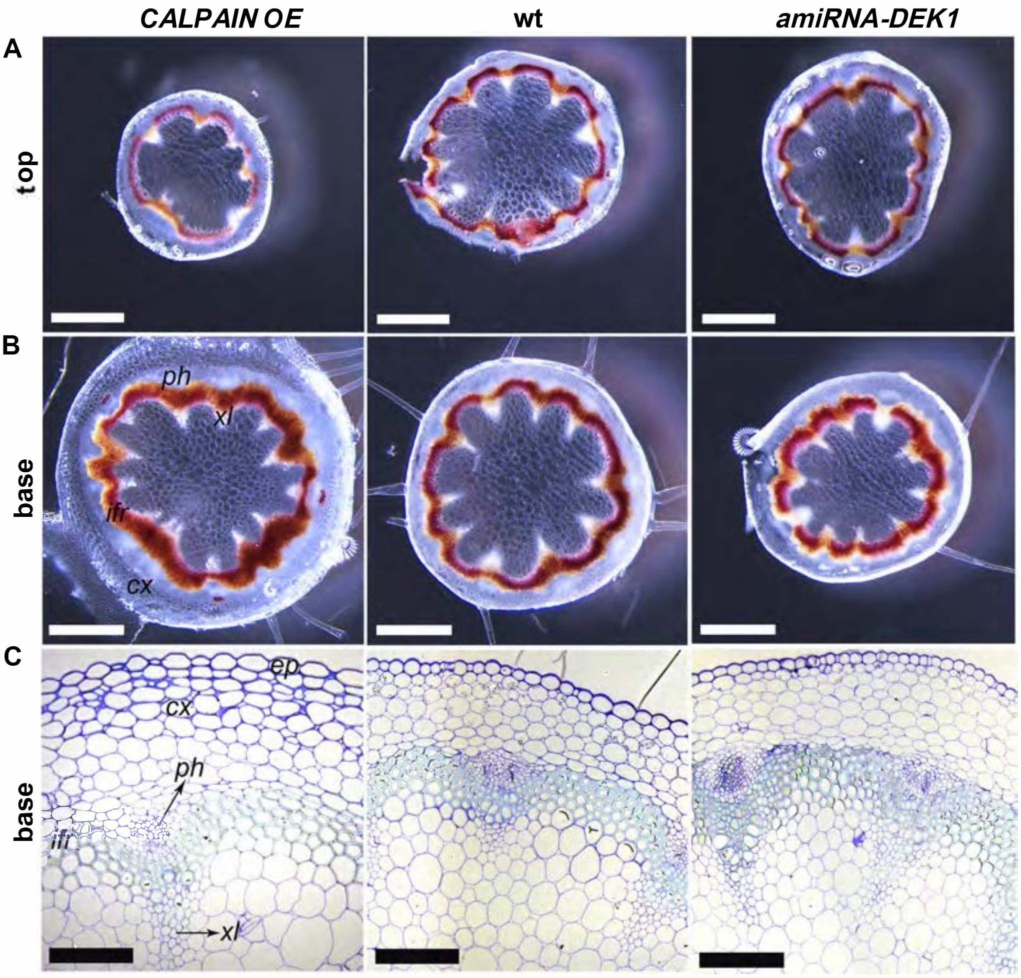

Supplementary Fig. S3. Light micrographs of cross-sections showing tissue organization at the stem base and 3 cm above the stem base of 4 month-old *CALPAIN OE*, wt, and *amiRNA-DEK1* stained with:

(A) and **(B)** Phloroglucinol-HCl stain taken from 3 cm above the base **(A)** and basal **(B)** part of the stem (as indicated in Supplemental Fig. S2). Compared to wt, increased and decreased basal stem diameter is observed in CALPAIN OE and amiRNA-DEK1, respectively **(B)** and decreased diameter in the region 3 cm above the base of CALPAIN OE stems **(A)** (scale bar= 500 µm).

(C) Toluidine blue staining shows increased cell size in epidermal, cortical, and pith cells of CALPAIN OE (scale bar= 250 µm). ep = epidermis; ex= cortex; ph = phloem; ifr = interfascicular fiber region; xi= xylem.

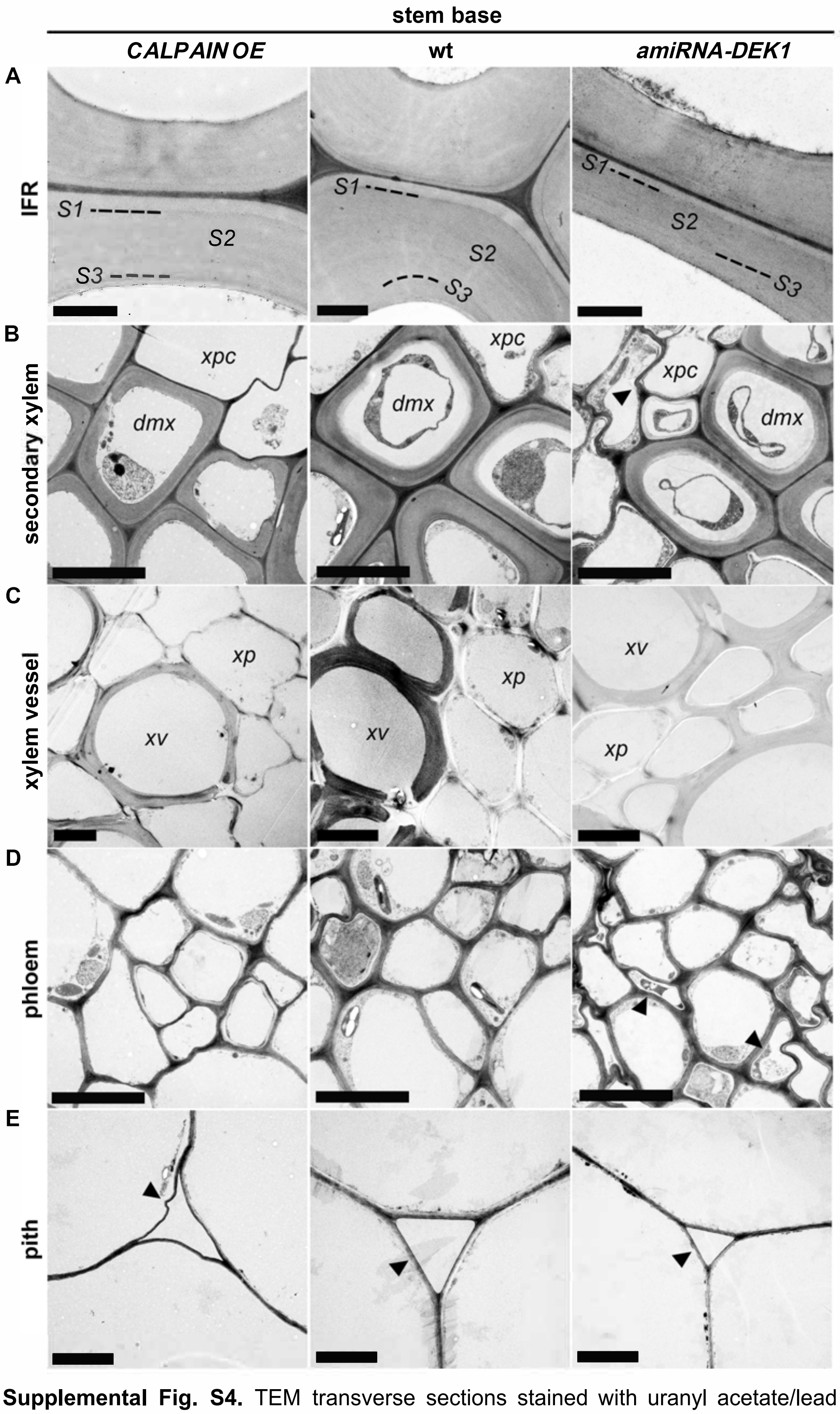

Supplemental Fig. S4. TEM transverse sections stained with uranyl acetate/lead citrate of the stem base from 4 month-old *CALPAIN OE*, wt, *amiRNA-DEK1* plants.

(A) Reduced IFR walls thickness is seen in *CALPAIN OE* and *amiRNA-DEK1*. *S1* = outer layer; *S2* = middle layer; *S3* = inner layer; scale bar = 1 μm. **(B)** to **(E)** morphology of secondary xylem **(B)**, xylem vessel **(C)**, phloem **(D)**, and pith **(E)** cells. Collapsed xpc **(B)** and phloem cells are seen in *amiRNA-DEK1* (arrowhead), while *CALPAIN OE* shows irregular shape of pith cell walls (arrowhead,**(D)**).

(xpc = xylary procambium; dmx = developing metaxylem; xv = xylem vessel; xp = xylem parenchyma; scale bar= 5 μm).

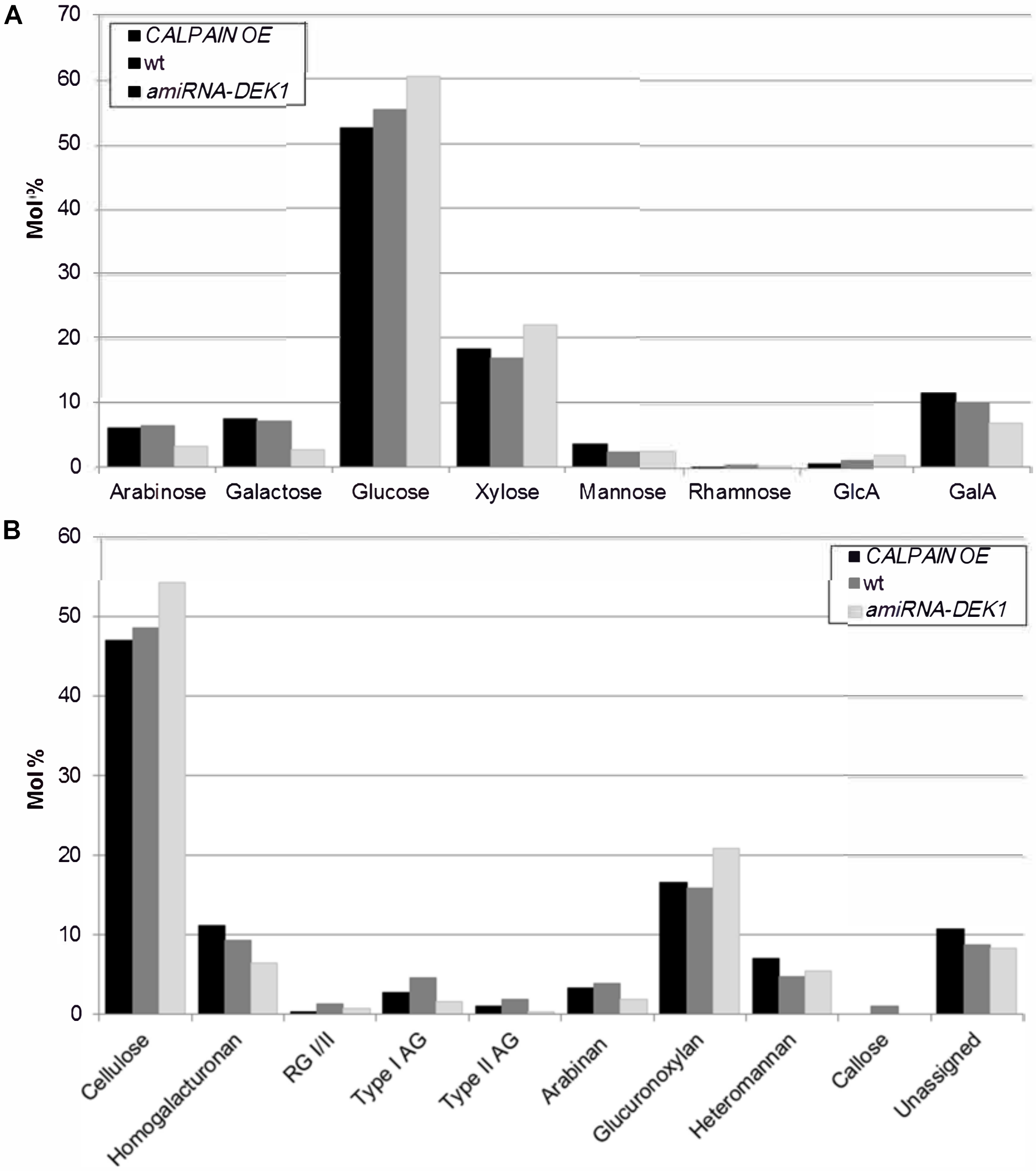

Supplemental Fig. S5. Analysis of cell wall monosaccharide and polysaccharide composition in the 3 cm basal region of 4 month-old *CALPAIN OE*, wt and *amiRNA-DEK1* stem.

(A) Monosaccharide analysis and **(B)** linkage analysis show no changes in wall composition across the lines.

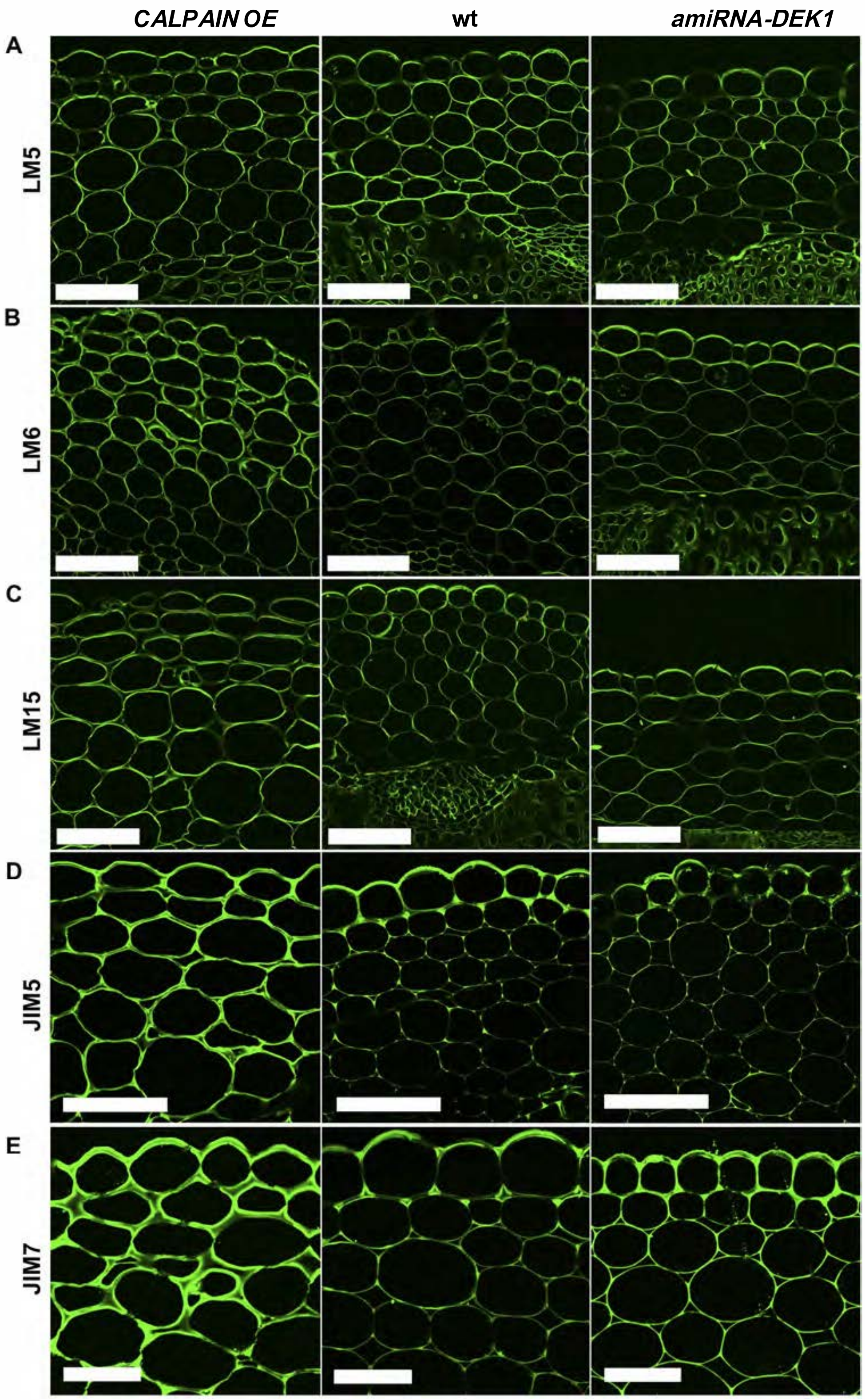

Supplemental Fig. S6. Detection of cell wall epitopes in the cross sections of the basal part of 4 month-old *CALPAIN OE*, wt, and *amiRNA-DEK1* the stems. **(A)** to **(C)** Immunofluorescence labelling shows no difference in labelling intensity of LM5 (galactan; **A**), LM6 (arabinan; **B**), and LM15 (xyloglucan; **C**) across the lines.

(D) to **(E)** Immunofluorescence labelling shows higher labelling intensity with JIM5 (low-DE HG; **D**) and JIM7 (high-DE HG; **E**) in *CALPAIN OE* compared to wt. (scale bar= 50 µm)

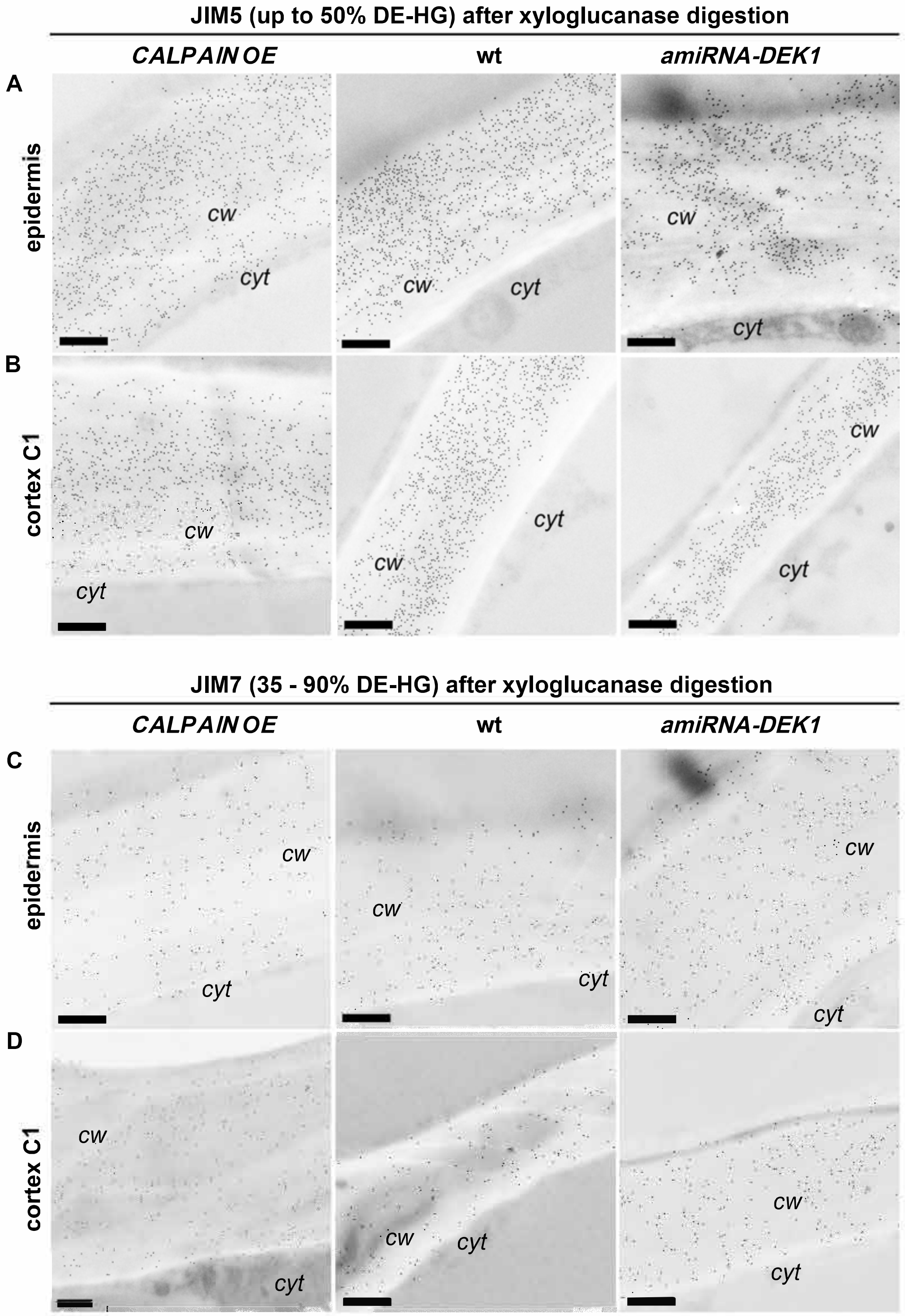

Supplemental Fig. S7. TEM immuno-gold labelling of pectin epitopes in the epidermis and cortex C1 periclinal walls from the transverse section of 4 month-old stem base.
(A) to **(B)** Labelling of JIM5 epitopes.
(C) to **(D)** Labelling of JIM7 epitopes.
No changes in gold density is observed across the lines.
(*cw* = cell wall; *cyt* = cytoplasm; *ml* = middle lamella; scale bar = 0.5 µm)

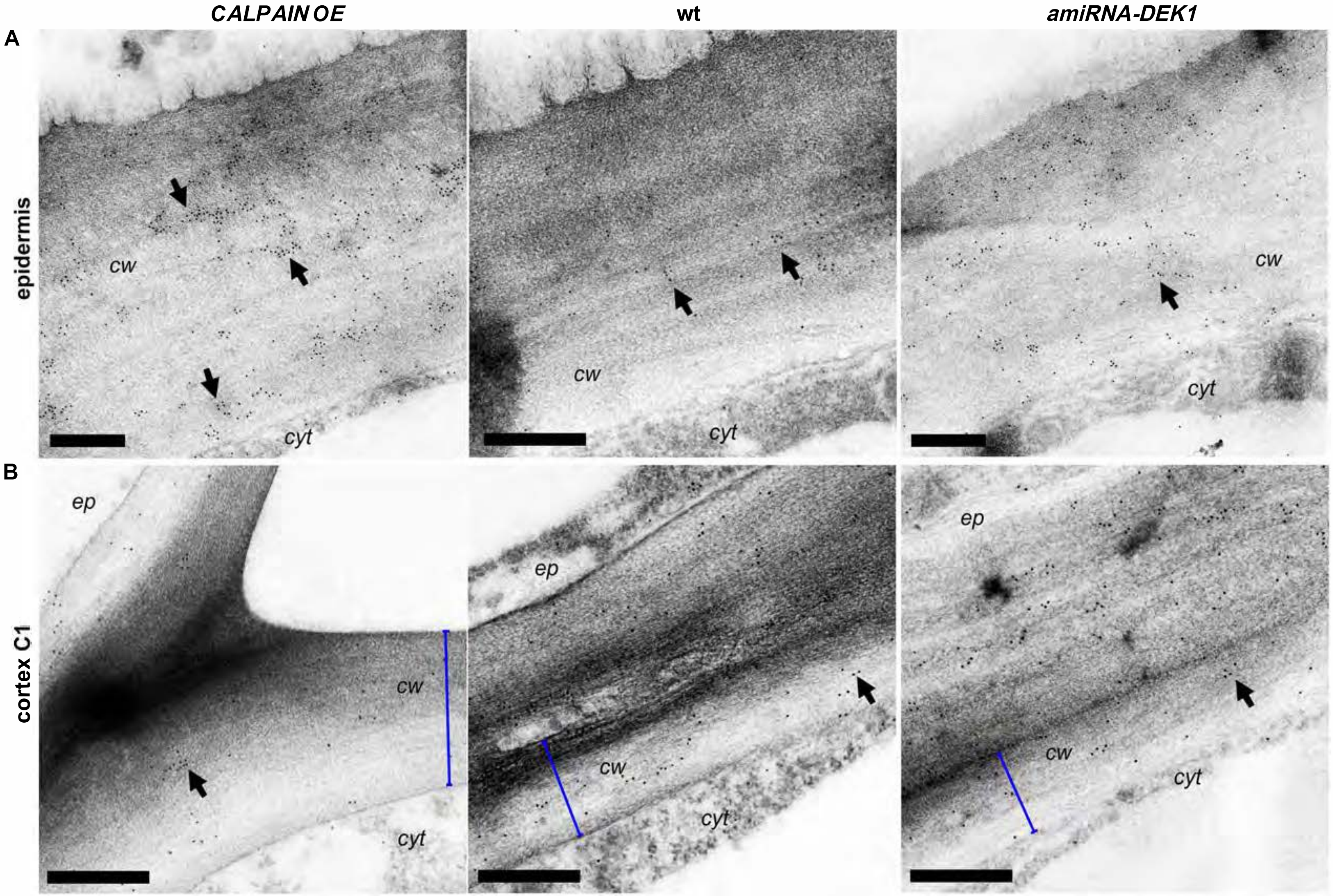

Supplemental Fig. S8. TEM immuno-gold labelling of cellulose epitopes by CBM3a in cross sections epidermal and cortex C1 (blue line) from 4 month-old stem base of *CALPAIN OE* and *amiRNA-DEK1*.

Increased gold labelling is seen in epidermal walls of *CALPAIN OE* (cw = cell wall; *cyt* = cytoplasm; scale bar = 0.5 µm).
